# Liquid Crystal Devices for Beam Steering Applications

**DOI:** 10.3390/mi12030247

**Published:** 2021-02-28

**Authors:** Rowan Morris, Cliff Jones, Mamatha Nagaraj

**Affiliations:** School of Physics and Astronomy, University of Leeds, Leeds LS2 9JT, UK; py13rm@leeds.ac.uk (R.M.); j.c.jones@leeds.ac.uk (C.J.)

**Keywords:** beam steering, lenses, adaptive optics, liquid crystals, polarisation gratings, liquid crystal alignment, gradient index devices, diffraction

## Abstract

Liquid crystals are valuable materials for applications in beam steering devices. In this paper, an overview of the use of liquid crystals in the field of adaptive optics specifically for beam steering and lensing devices is presented. The paper introduces the properties of liquid crystals that have made them useful in this field followed by a more detailed discussion of specific liquid crystal devices that act as switchable optical components of refractive and diffractive types. The relative advantages and disadvantages of the different devices and techniques are summarised.

## 1. Introduction

Liquid Crystals (LCs) are remarkable electro-optic materials most notably utilized in Liquid Crystal Displays (LCD) [1,2], but increasingly in adaptive optical components. The development of efficient and switchable liquid crystal beam steering devices is a major area of research, as it has a wide range of potential applications, such as head-worn displays [3,4,5], smart contact lenses [6,7,8,9], solar cells [10], holography [11] and smart windows [12]. LCs are used widely in the field of adaptive optics due to their refractive index anisotropy, the variety of alignment methods that are available, and the ability of liquid crystals to reorient when subjected to relatively low energy stimuli. Over the decades, a broad range of liquid crystal beam steering devices has been created utilizing both refractive and diffractive optics [13,14,15,16,17,18,19,20,21,22]. Various methods have been employed in creating new LC beam steering devices, depending on the requirements for the proposed application. Often this has led to new research directions, although common goals have been to improve the efficiency, switching speed, aperture size, maximum deflection angles, continuously variable angle, polarisation-independence, ease of fabrication and operation, and bi-stability. This article provides an overview of some of the properties of liquid crystals relevant for beam steering applications and examines the general approaches employed to create such devices. This will include a discussion of the operating principles, performance, advantages, and disadvantages. 

### 1.1. Static Properties of Nematic Liquid Crystals 

The majority of LC beam steering devices use Nematic Liquid Crystals (NLC), which have long-range orientational order only. This order is defined relative to the average orientation direction of LC molecules, known as the director, ***n***. The nematic phase usually has the optical properties of a uniaxial crystal (***n*** = −***n***), where the refractive index (*n_ref_*) can be written as

(1) $$n_{ref}=\left(\begin{array}{ccc} n_{o} & 0 & 0\\ 0 & n_{o} & 0\\ 0 & 0 & n_{e} \end{array}\right)$$

or

(2) $$n_{ref}\left(\psi \right)=\frac{n_{o}n_{e}}{\sqrt{n_{o}^{2}cos^{2}\psi +n_{e}^{2}sin^{2}\psi }}$$

where *n_o_* and *n_e_* are the ordinary and extraordinary refractive indices, respectively, and *ψ* is the relative angle between the incident light’s electrical polarisation vector and the director ***n***.

The optimum ***n*** field of a NLC can be found by minimizing the system’s free energy (*F*_NLC_). This is made up of several contributing components, such as the material elasticity, alignment and externally applied fields. In the absence of external fields, *F*_NLC_ is written,

(3) $$F_{\mathrm{NLC}}=F_{K}+F_{\mathrm{AL}},$$

where *F*_K_ and *F*_AL_ are contributions to the free energy by the nematic field’s elasticity and alignment, respectively. In the absence of defects, *F*_K_ can be written as a summation of three primary deformations that ***n*** can undergo, each of which has an associated elastic constant (*k_ii_*) [23,24,25],

(4) $$F_{K}=\frac{1}{2}\int_{V}\;[k_{11}\;{\left(\nabla .n\right)}^{2}+k_{22}{\left(n.\nabla \times n\right)}^{2}+k_{33}{\left(n\times \nabla \times n\right)}^{2}dV,$$

where, *k*_11_, *k*_22_ and *k*_33_ are elasticities associated with splay, twist and bend deformations, respectively. *F*_AL_ is quantified by summating deviations from the optimum alignment on the enclosing surface (*Σ*). *F*_AL_ is written as two parts associated with azimuthal (*ϕ)* and radial (*ω*) deviations of ***n*** with respect to *Σ*’s normal

(5) $$F_{\mathrm{AL}}\left(\omega ,\;\phi \right)=F_{\mathrm{AL},\omega }\left(\omega \right)+F_{\mathrm{AL},\;\phi }\left(\phi \right),$$

where

(6) $$F_{\mathrm{AL},\;\omega }\left(\omega \right)=\frac{1}{2}\int_{\Sigma }W_{\omega }sin^{2}\left(\omega -\omega_{\mathrm{AL}}\right)d\Sigma$$

and

(7) $$F_{\mathrm{AL},\;\phi }\left(\phi \right)=\frac{1}{2}\int_{\Sigma }W_{\phi }sin^{2}\left(\phi -\phi_{\mathrm{AL}}\right)d\Sigma .$$

Here *ϕ*_AL_ and *ω*_AL_ are the optimum orientations of the director at the surface, while *W_ϕ_* and *W_ω_* are their anchoring energies [26,27]. Practically, the alignment of liquid crystals can be achieved with a wide variety of methods, such as mechanically rubbed thin films [28,29,30,31], the directed evaporation of thin films [32], topographical relief structures [33,34,35,36], optically patterned films (photoalignment) [37,38,39,40,41] and stacked alignment layers [42,43]. Such alignment acts to give ***n*** some desired initial state and is highly relevant to many beam steering devices.

### 1.2. Dynamic Properties of Nematic Liquid Crystals 

In order to create switchable beam steering devices, the optical properties of NLCs are changed by applying an external field; electric [44], magnetic [44,45], elastic [46] and viscous flow [15,47] can all be used. The exact nature of reorientation of the director under an external field depends on the sign, magnitude and frequency of the applied field and the initial director configuration with respect to the field direction. Generally, the reorientation occurs due to the field’s coupling to a relevant anisotropic property of the NLC phase to reduce *F*_NLC_. 

In LC beam steering, electric fields are commonly utilized to reorient the NLC. This process is known as an electric Fréedericksz transition and is caused by the liquid crystal director reorienting to resist the applied field maximally [25,44,45]. The electric field couples to the materials’ dielectric anisotropy, *Δε*, in this case, where

(8) $$\Delta \varepsilon =\varepsilon_{∥}-\varepsilon_{⏊}.$$

Here *ε*_‖_ and *ε*_⏊_ are the permittivity parallel and perpendicular to the director, respectively. The application of a field, therefore, adds an electric field term (*F*_E_) to *F*_NLC_, so *F*_NLC_ becomes [44]

(9) $$F_{\mathrm{NLC}}=F_{K}+F_{\mathrm{AL}}+F_{E}$$

where

(10) $$F_{E}=\frac{1}{2}\int_{V}\Delta \varepsilon \varepsilon_{0}{\left(E\cdot n\right)}^{2}dV.$$

Usually, the time required for director reorientation to occur (*τ*_on_) depends on the strength of the applied field and material parameters; meanwhile, the time to relax back to the original state (*τ*_off_) is only dependent on the material elasticity. For electric fields, these can be written as [48],

(11) $$\tau_{\mathrm{on}}\propto \frac{\gamma }{\varepsilon_{0}\Delta \varepsilon E^{2}-\frac{K}{d^{2}}}$$

and

(12) $$\tau_{\mathrm{off}}\propto \frac{d^{2}}{K},$$

respectively. Where *d* is the thickness of the liquid crystal layer and *K* and *γ* elastic and viscous constants chosen to be appropriate for the device configuration.

### 1.3. Refractive and Diffractive LC Beam Steering Devices 

As switchable uniaxial crystals with the above discussed physical properties, NLCs are well suited to create a wide variety of switchable beam steering devices. These will be the focus of the remainder of this review. Firstly, Section 2 will discuss refractive devices, while Section 3 will discuss those based on diffraction. In this work, we class any device where the first diffractive order is less than 0.5° from the central order as refractive, while those where it is larger than this is diffractive. Taking a typical wavelength of optical light (500 nm), this causes devices where feature sizes are larger than around 60 μm to be refractive, while causing those that are smaller to be diffractive. Both Section 2 and Section 3 will briefly describe how refractive and diffractive effects can be utilized for beam steering, respectively, and then discuss specific LC-based approaches. Finally, Section 4 will summarise the findings and overview the advantages and disadvantages of the different techniques. 

## 2. Refractive NLC Optical Devices 

### 2.1. Refractive Optical Components 

**Figure 1:**
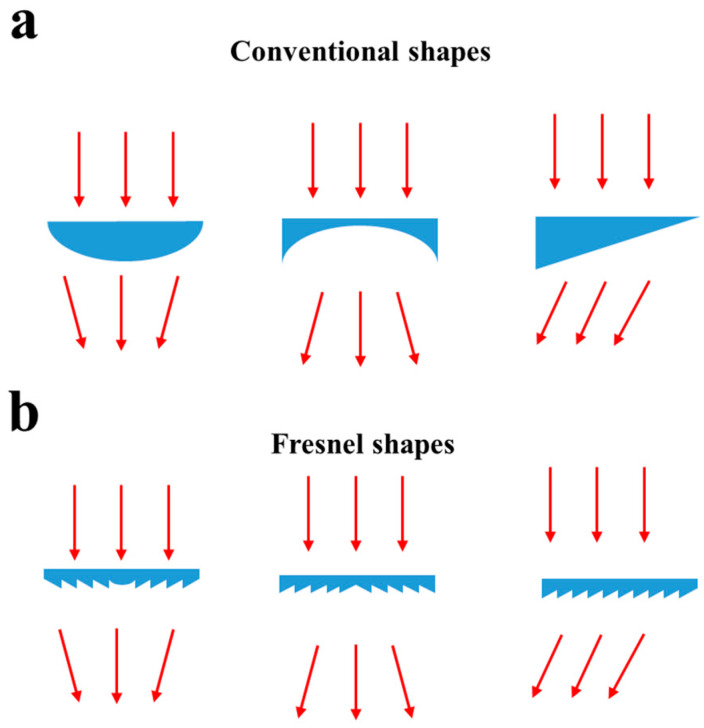
(**a**) Shows three examples of static beam steering or lensing devices. Here left and middle figures show focusing and defocusing lenses, respectively. The right-hand side shows a simple deflecting prism. (**b**) Shows the refractive Fresnel equivalents of the structures shown above.

Before considering LC refractive beam steering devices, it is useful to consider standard static refractive optical components, such as glass lenses and prisms. These are well-known to operate through the Poynting vector’s reorientation at a surface where the refractive index changes. This is expressed within Snell’s Law as

(13) $$\frac{n_{1}}{n_{2}}=\frac{sin\theta_{2}}{sin\theta_{1}}$$

where *n*_1_ and *n*_2_ are refractive indices on either side of the interface, and *θ*_1_ and *θ*_2_ are the corresponding angles. Figure 1a shows several devices that utilize Snell’s Law to focus, defocus and deflect optical beams. A well-known method for reducing such devices’ thickness is by resetting the feature height to create a refractive Fresnel structure (Figure 1b). These can be made to approximate the optical effects of the thicker non-Fresnel components, provided the features are not so small that significant diffraction occurs [49]. 

Of particular importance to describe NLC optics are Gradient Index (GRIN) effects. Here, instead of the sudden changes in refractive index, described by Equation (13), a smooth change in refractive index is experienced by light. For this, the continuous differential form of Snell’s Law must be used [49,50,51],

(14) $$\frac{\partial^{2}x}{\partial z^{2}}=\frac{1}{n}\cdot \frac{\partial n}{\partial x}.$$

Here, a uniform GRIN will bend light towards the higher refractive index, as the wavefront speed is reduced. This can be utilized to create static lensing, waveguiding and beam steering structures [49,50,51,52]. The static GRIN devices can be fabricated using several methodologies [49,53], and are used widely in optical storage and communications [49,50,54,55].

### 2.2. Uniaxial Optical Components 

As discussed in Section 1.1 and Section 1.2, NLCs are birefringent materials, the optic axis of which is aligned along, ***n***, which can be controlled using both boundary conditions and externally applied fields. From Equations (13) and (14), this ability to change the refractive index can create switchable beam steering and lensing components using liquid crystals. These devices are generally polarization-dependent due to the NLC being uniaxial, where transmitted rays of orthogonal polarisation are split around the optical axis. 

**Figure 2:**
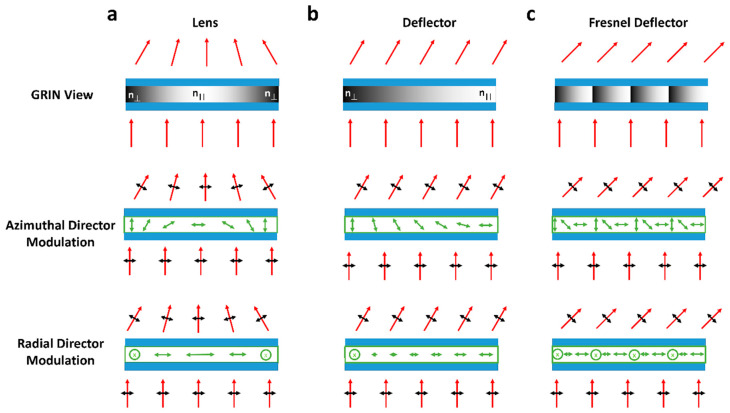
GRIN structures required for beam steering and lensing devices, with how they can be achieved for linearly polarised light by controlling either the radial or azimuthal orientation of ***n***. (**a**) and (**b**) show a simple lens and deflector which will have relatively small gradient indexes. (**c**) Shows how (**b**) could be altered with a Fresnel structure to increase the gradient in refractive index for more significant optical deviation.

If the director continually rotates in space, each polarization of light will experience a different GRIN. If this can be controlled, a switchable beam steering device can be created. Examples of desirable GRIN structures to create a focusing lens or beam deflector are shown in Figure 2a,b. The figure also shows appropriate LC director configurations to achieve such optical effects, where both azimuthal and radial director modulation can be utilized.

In practice, when creating refractive liquid crystal beam steering devices, the small difference between *n_o_* and *n_e_* (typically around 0.2) and LC devices, being relatively thin (*d* < 20 μm) causes only small deviations to outgoing optical beams. An LC refractive Fresnel structure can be used to increase this deflection angle. Here, by resetting the director orientation, a larger value $\frac{\partial n}{\partial x}$ is created, leading to increased optical deviation [56] (Figure 2c). 

### 2.3. Geometric Prisms and Lenses 

**Figure 3:**
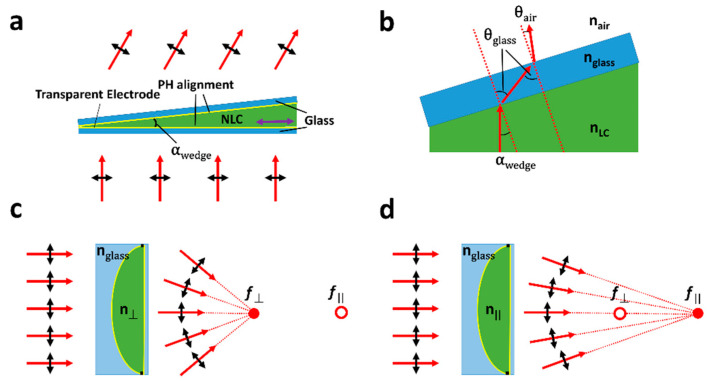
Shows “geometric” LC lenses and beam steerers. (**a**) Shows a simple LC wedge device, filled with a positive *Δε* NLC. Here, the alignment is planar homogeneous, and the purple arrow shows the rubbing direction. When a large voltage is applied across the NLC, the refractive index experienced by light will change, leading to switching output angle. (**b**) Shows a zoomed-in schematic of this at the NLC–glass–air interface. (**c**,**d**) show the same effect operating for a lens. Here the LC adopts a lens shape in a glass cavity, which when the refractive index switches will change the focal point of rays from *f*_⏊_ to *f*_‖_.

The simplest liquid crystal beam steering devices can be made up of a geometrically shaped lens or prismatic structure. Here, the refractive index and so the focus or deflection angle can be changed by applying some external field. Figure 3a shows an example beam deflector which operates using this principle. The emergent angle of the ray of light can be shown using Equation (13) as

(15) $$sin\theta_{air}=\frac{n_{\mathrm{glass}}}{n_{\mathrm{air}}}sin\theta_{g}=\frac{n_{\mathrm{LC}}}{n_{\mathrm{air}}}sin\alpha$$

where *n*_glass/air/LC_ are the refractive indices of glass, air and the LC, respectively, and the angles *θ_air_* and *θ*_glass_ are shown in Figure 3b. For a typical NLC at optical wavelengths (*n*_o_ = 1.5, *n_e_* = 1.7) and in a highly wedged device (α_wedge_ = 2°) the change in the emergent angle is around 0.5°, which is significantly smaller than that which is desired for many beam steering applications. Indeed, when a similar prism device was published by Love et al., in 1994 [19], it was found that in a 10 μm device steering of less than 0.01° occurred. Sato used a similar approach with a much thicker NLC layer to create a lens in 1979 [13]. Here, in a device similar to that shown in Figure 3c,d, a lens with a switchable focal point (*f* = 160 to 220 mm) was demonstrated. Again, this switchable behaviour is insufficient for many proposed applications but demonstrates one of the first LC-based beam steering devices. 

In addition to the small switchable angles, geometric LC beam steering devices have further drawbacks due to the thickness of the layer of LC (*d*) required. Large *d* leads to disadvantages of slow switching, decreased transmissivity and increased likelihood of forming defects [13,57]. In the work by Titus et al. in 1999 [58], many devices, similar to that shown in Figure 3a, were put into series to increase steering angle and approximate continuously switchable behaviour. However, this stacking led to significant drawbacks in optical transmission, device compactness and complexity of addressing.

One area where geometric LC devices have been widely applied more recently is switchable contact lenses for presbyopia correction. Here, due to the relatively modest focusing powers required (less than 2.5 dioptres [7,8]) and the natural curvature of the eye, geometric devices are potentially well-suited for smart contact lens applications [9]. 

### 2.4. Refractive Fringing Field Devices

#### 2.4.1. Beam Deflectors 

**Figure 4:**
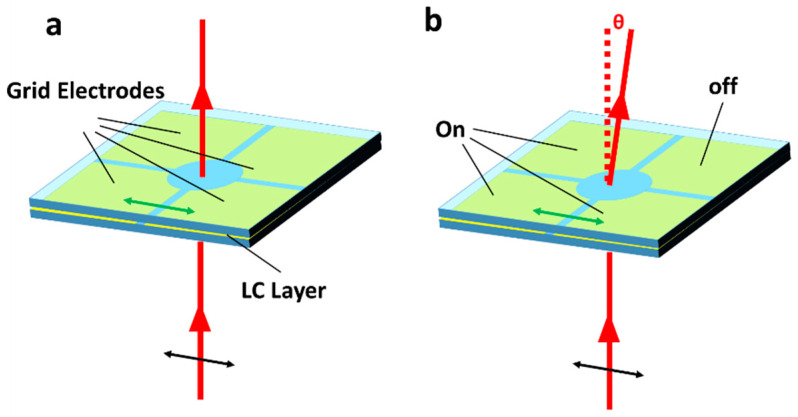
Gradient index refractive beam deflector. Here, through controlling voltages applied to each electrode, deflection of an optical beam can be achieved. (**a**) The device in the absence of applied voltage, where the beam passes through without deviation. (**b**) The device with several electrodes with voltage applied, inducing a gradient index which deviates the transmitted beam. The rubbing direction is marked as the green double-headed arrow on the device, and the input polarisation as the black arrow.

An alternative approach to create beam steering devices using NLCs is to utilize electric field fringing. Here, due to the Electro-Optic effect, electric field fringing at the edges of electrodes allows GRIN structures, such as those shown in Figure 2, to be formed. In the absence of Fresnel offsets, such devices have limited values of $\frac{\partial n}{\partial x},$ meaning that the maximum deflection angles tend to be small. Additionally, the width of the device (*w*) is inversely related to $\frac{\partial n}{\partial x},$ meaning that wider aperture devices of the same *d* steer to smaller angles. In 1997, Masuda et al. [59] and, later in 2006, Ye et al. [60], created and assessed four-electrode grid-patterned devices, which allowed for switchable refractive beam deflection. Here, the four electrodes allowed for beam deflection in four directions, due to inducing a GRIN (see Figure 4). For the devices presented by Ye et al. [60], where the aperture was ~12 mm^2^ and the LC layer was 130 μm thick, continuous steering to a distance of approximately 150 μm at 100 mm (~*Δθ* = 0.1°) was achieved. Such a small deflection angles, even in relatively thick devices, have led to “refractive fringing field device” research to generally focus on the creation of lenses, where they can continually focus from infinity to some finite focal point. The rest of this section will focus on the various methods that are used to create such devices. 

#### 2.4.2. Lenses

**Figure 5:**
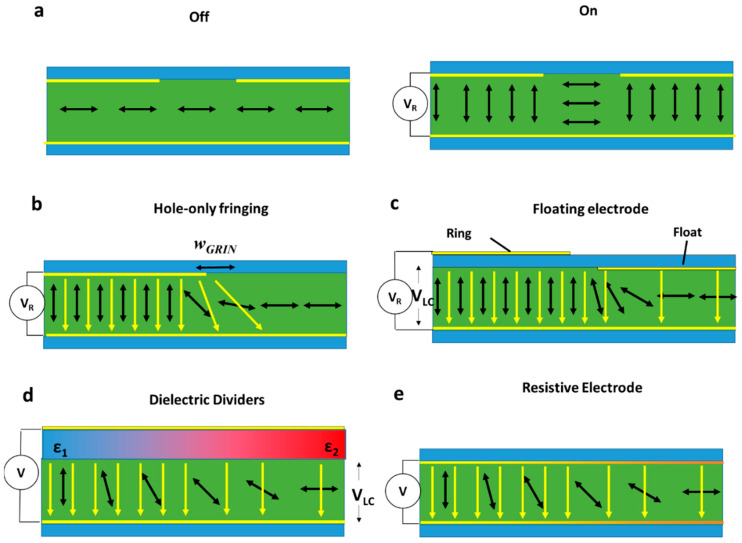
Methods of creating and improving GRIN devices with planar electrodes. (**a**,**b**) Shows a hole-patterned lens which is either on or off. (**c**) Shows a zoomed-in schematic, where the fringing field causes the director to reorient over a width w_GRIN_. (**c**–**e**) Show methods of increasing w_GRIN_ through additional device features. (**c**) Shows an additional “floating” electrode. (**d**) Shows this combined with a variation in the permittivity of the substrates, smoothly reducing the applied field. (**e**) Shows the effect of resistive electrodes, where yellow electrodes indicate full charging, while orange represents no charge.

The first NLC fringing field lens was created by Nose et al. in 1992 [61]. This was a so-called “hole-patterned” device, where the fringing field was induced through removing a 700 μm circular part of the electrode (see Figure 5a). Such hole-patterned lenses are limited for three main reasons, firstly the width over which the GRIN exists (*w*_GRIN_ in Figure 5b) is small, limiting lens size. Secondly, the profile of $\frac{\partial n}{\partial x}$ is not smooth, due to the sudden director reorientation at the Freedericz transition. Thirdly, as the aperture size becomes bigger, the value *w*_GRIN_ remains the same, while the value $\frac{\partial n}{\partial x}$ decreases. This significantly limits the focussing power and efficiency of wide aperture (>1 mm^2^) hole-patterned devices. 

To address the relatively small value of *w*_GRIN_, several approaches have been employed. Firstly, a further “floating” electrode can be added (see Figure 5c) [62,63,64,65,66]. Here, an extra electrode is added to the lens’ centre, which acts to redistribute the fringing field further from the hole-patterned electrode. This has been shown to significantly smooth the phase retardation profile of the lens, allowing higher quality lensing [62,63]. However, as shown in Figure 5c, it also separates the outer ring electrode from the LC by a glass layer. This separation leads to a significant drop in electric potential across the substrate (*V*_sub_), reducing that over the NLC (*V*_LC_). *V*_LC_ can be calculated using,

(16) $$V_{\mathrm{LC}}=\frac{V_{R}}{1+\frac{d_{\mathrm{sub}}\varepsilon_{\mathrm{LC}}}{d_{\mathrm{LC}}\varepsilon_{\mathrm{sub}}}},$$

where *V*_R_ is the potential applied to the ring, $d_{\mathrm{sub}}$ and $d_{\mathrm{LC}}$ are the thicknesses of the substrate and NLC layer, and $\varepsilon_{\mathrm{sub}}$ and $\varepsilon_{\mathrm{LC}}$ are the electric permitivities. The reduction in potential leads to high voltages being required to drive such devices where, for example, in a device built on thin (100 μm) glass ($\varepsilon_{\mathrm{sub}}=5)$ with a LC of typical permittivity ($\varepsilon_{\mathrm{LC}}=10)$ and thickness (5 μm), *V*_LC_ is only ≈2% of $V_{R}$. 

A second approach is to smooth the director reorientation using specialized substrates which continuously change value $\varepsilon_{\mathrm{sub}}$ across them. From Equation (16), it is clear that, by doing so, *V*_LC_ can be controlled. An example of such a device based on this dielectric dividing principle is shown in Figure 5d, where varying $\varepsilon_{\mathrm{sub}}$ from $\varepsilon_{1}$ to $\varepsilon_{2}$ across the device, *V*_LC_ can be smooth. Practically, such substrates have been achieved with various techniques; for example, building the substrate of two materials, the relative thicknesses of which modulate *ε*_sub_ [67,68]. Alternatively, NLCs themselves can form this dielectric structure where, for example, Polymer Dispersed Liquid Crystals (PDLC) can be arranged to adopt a structure of the required permittivity and then cured into place [69,70]. Both have excellent optical properties, having the advantage of only one electrode; however, similarly to floating electrode devices, they require large driving voltages. 

Improved performance can be accomplished using highly resistive electrodes. Here, due to a finite time being required to fully polarise the electrodes, different electrode areas have different charge distributions, reducing voltages across the NLC further away from the voltage source [71,72,73,74,75] (Figure 5e). These devices are naturally highly frequency-dependent due to such devices being reliant on a finite electrode charging time, giving an advantageous extra control mechanism, but also requiring extra considerations to be taken in electrode design and choice of LC materials. 

These methods of increasing *w*_GRIN_ can often be combined to create further optimized hybrid devices [62,76,77,78,79]. Several reviews of such techniques for lensing applications have been published [9,74,80]. 

### 2.5. Alignment Controlled Refractive Beam Steering 

#### 2.5.1. Stacked Alignment Layers 

A different approach to creating NLC beam steering components is through utilizing alignment to force ***n*** to adopt some beam steering configuration in the absence of other stimuli (see Figure 2). This structure can then be removed by applying a large external field, which reorients the optic axis of the NLC through a Fréedericksz transition. The methods involved in such approaches have previously been the subject of several review articles [40,81]. 

**Figure 6:**
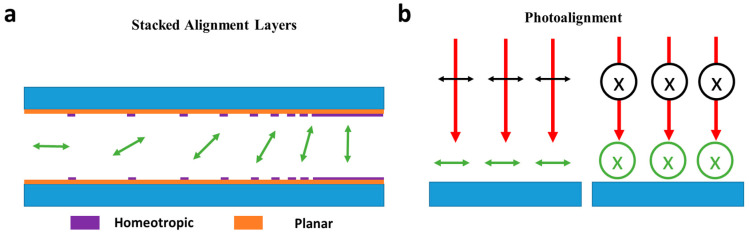
Schematic of variations in director orientations due to different alignments (**a**) Stacked alignment layers can smoothly vary pretilt angle through scribing through the top alignment layer to reveal the alignment layer underneath. Here, the density of scribing will increase planar alignment, allowing for the continuous variation of azimuthal angle. (**b**) Shows an example of photoalignment, where the radial orientation of the alignment can rotate when exposed to an optical field of a particular linear polarisation.

One approach to controlling ***n*** is using Stacked Alignment Layers (SALs). Here, two or more aligning layers are deposited on top of one another and the top one removed selectively so that the lower layer also partially induces alignment on the LC (Figure 6a) [42,82,83]. An early example of using this method was Patel et al. in 1991 [84], where a refractive Fresnel lens with an aperture of several mm was constructed through orthogonal rubbing of two photopolymers, and selectively removing the upper layer to create the pattern. Due to orthogonal rubbing, the lens was close to achieving polarization independence and had a maximum efficiency of around 35% for both linear polarizations. In 2011, Tseng et al. [82] utilized SAL techniques to control the director’s azimuthal angle fairly continuously through UV dosage modulation to create a SAL lens. Here a 19–400 cm variable focal point was achieved from a 2 mm aperture. More recently, for further localized control, ion-beam lithography was used to reduce the minimum feature size [85,86]; however, such devices have not yet been applied to create beam steering effects. 

#### 2.5.2. Photoalignment 

A different method to create alignment-based beam steering devices is photoalignment (Figure 6b) [41,87]. Here, by exposing the alignment layer to a polarised optical source, the direction of alignment can be forced to vary either radially or azimuthally [40,81,87]. This can then be utilized to create either GRIN lenses and beam deflectors [88,89,90]. Bezruchenko et al., published an NLC lens based on this technique [88], where photoalignment was used to control NLC pretilt, creating a continuous lensing structure. The resulting device had a continually variable focal length from 47–700 cm with a relatively small driving voltage change of 0–10 V. This device also demonstrated limited beam steering of 0.14° if a beam was passed through an off-centre part of the lens. 

### 2.6. Electronically Addressed Refractive Spatial Light Modulators

An alternative method for creating the GRIN structure required for beam steering is through using direct pixelated electrode modulation. Here, devices consisting of finely spaced electrodes, which can be individually addressed with different voltages to create the desired director structure (see Figure 2a) are created. A patent on this was filed by Canon in 1982 [91], and a proof of concept for such devices was given by Kowel et al. in 1984 [92]. Due to the increased availability of lithographic techniques, numerous electrode designs can be created, including the concentric ring electrodes design [93,94]. Increasing in complexity, Electronically Addressed Spatial Light Modulators (EASLM) can now be used to apply arbitrary electric fields to NLCs to create desired phase structures [14,95,96,97,98,99,100,101,102,103,104,105,106,107]. These operate through applying fields to many pixelated transistors, each of which can be activated independently in a similar manner as a flat panel display [48]. 

**Figure 7:**
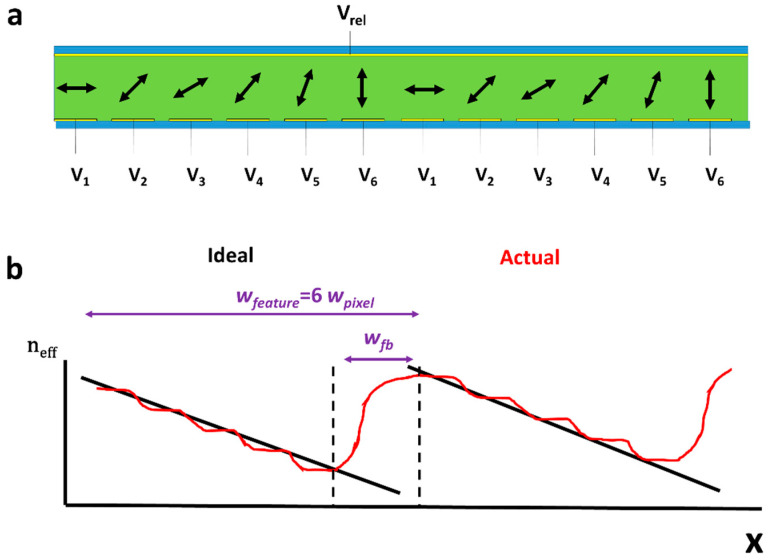
Shows an EASLM creating a prism structure. (**a**) Shows a director configuration with one Fresnel type offset. This doubles the value $\frac{\partial n}{\partial x}$ compared to a completely continuous system, allowing wider beam deflection. (**b**) Demonstrates the device’s limitation due to finite *w*_pixel_ and *w*_fb_ sizes, which prevent the perfect structure in different ways. Their effects will increase with smaller values *w*_feature_.

Both multiple electrodes and EASLMs can be used to create refractive or diffractive (see Section 3.5) beam steering structures. In both, the key parameters to create high-quality optical structures are the size of individual pixels (*w*_pixel_), the range of different director reorientations/grey levels which can be achieved (*N*_grey_) and the width of the “flyback region” (*w*_fb_, where the director resets to zero to create the desired Refractive or Diffractive Fresnel offset [106]). *w*_pixel_ and *w*_fb_ are illustrated in Figure 7b, where it can be seen that both, being finite sizes, reduce the quality of the refractive index profile. The smallest pixel sizes currently achieved when the device is placed upon a silicon backplane (Liquid Crystal on Silicon, LCoS), are around 5 μm [104,108]. Due to silicon’s high reflectivity, such LCoS devices can only operate in reflection mode, whereas Liquid Crystal on Poly Silicon (LCoPS) can work in transmission mode leads to larger minimum pixel sizes [21,109]. The optical loss in efficiency associated with *w*_fb_ can be approximated as [21,22,102,106],

(17) $$\eta_{\mathrm{fb}}={\left(1-\frac{w_{\mathrm{fb}}}{w_{\mathrm{feature}}}\right)}^{2}$$

where *w*_feature_ is the width of the feature between resets (Figure 7b). 

When EASLMs are utilized to create refractive beam steering structures, the prism structure width (*w*_feature_, see Figure 7b) tends to be much greater than *w*_fb_. This leads to small losses; however, it also leads to small values $\frac{\partial n}{\partial x},$ resulting in limited beam deflection [102,106]. A detailed report on EASLMs as refractive beam steering was published by Mcmanamon et al. in 2009 [106]; here, it was reported that 99% efficiency was possible at 0.1° deflection, which reduced to 90% at 1°. This reduction is due to wider angles requiring more Fresnel offsets, leading to losses through diffraction [101,106] (diffractive EASLM devices are discussed in Section 3.5). 

### 2.7. Optically Addressed Refractive Spatial Light Modulators

An alternative mechanism to address individual pixels is through Optically Addressed Spatial Light Modulation (OASLM). Here a “writing beam” is used to create a pattern which leads to a reorientation of the NLC. One way this can occur is by optically inducing a change in the resistive properties of electrodes. Here, the electrodes are designed to change their resistivity when exposed to the writing beam, allowing a spatially dependent voltage (shown in Figure 5e) to be achieved [110,111]. Alternatively, the writing beam can directly reorient specially designed alignment layers [112] or NLC materials [113,114,115] to create the same effect. The drawbacks of such devices usually lie in the relatively bulky and complicated setups required for the writing beam to reorient the NLC. Additionally, relatively thick layers of NLC are required for wide steering due to practical difficulties using the technique to create Fresnel offsets [115]. 

### 2.8. Optical Waveguides 

**Figure 8:**
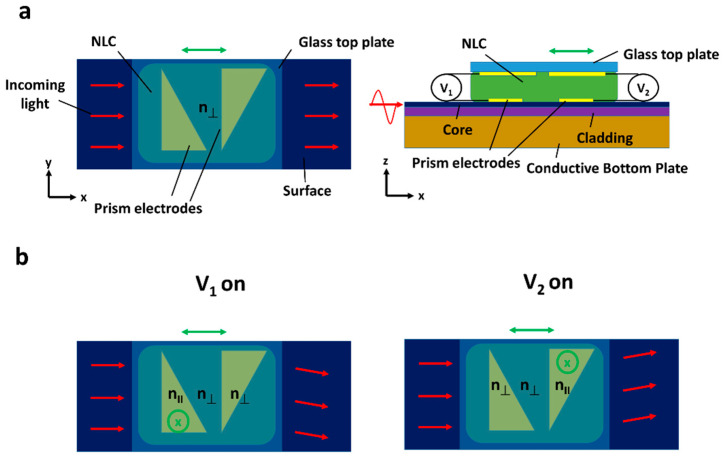
Shows an example of a liquid crystal waveguiding device. Here, light (red arrows) passes through the fibre core, and the produced evanescent wave interacts with the experienced refractive index of the LC in contact. This can be used to steer the ray as the LC can adopt a GRIN structure due to the electric field’s application. (**a**) Shows the device schematic with key features. (**b**) Shows its mode of operation, where the two voltages can be used for deflection to different directions.

GRIN devices are often limited by the LC layer’s thickness the light passes through (typically being less than 20 μm). This reduces the distance over which GRIN effects can deviate the transiting light, reducing the outgoing steering angle. One way to address this is to pass the light through the NLC parallel to the substrates and modulate over distances >1 mm. Early designs for such devices were described as early as 1977, where the aim was instead to measure optical losses through the NLC [116]. However, later on, the idea of light laterally passing through the LC layer was combined with static optical fibre elements to create NLC waveguides (see Figure 8) [117,118]. Here, the NLC controls the relative angle of the outgoing beam through modulation of the evanescent wave. These devices are highly successful in wide-angle (>80° continuous steering) and high efficiency (>80%) refractive LC beam steering [119,120]. However, such optical waveguides do have limitations, particularly in aperture size as light passes through the fibre-optic core, which typically is around 20 μm, making the technique unviable for several applications. 

### 2.9. Polarisation Independent Devices

NLCs being uniaxial crystals is a limitation in NLC beam steering devices as they often only act on a single input polarization (see Section 2.2), limiting efficiency to 50% for unpolarised light. Attempts to address this limitation usually use three main approaches. Firstly, through including two beam steering structures, one targeting each orthogonal polarisation. For example, a refractive Fresnel structure can be made for both polarisations, making the beam steering component polarization-independent [84,121,122,123]. Secondly, using a twisted nematic device. As the twisted nematic equally affects orthogonal components, polarization-independent lensing has been achieved [66,124]. Thirdly, more exotic LC phases can be used (discussed in more detail in Section 4.4). 

## 3. Diffractive NLC Optical Devices 

### 3.1. Raman-Nath Diffractive Optical Components 

An alternative to refractive devices are those based on diffraction. Here, LC beam steering devices operate through inducing a phase shift (*ϕ*) on transmitted light, which then constructively diffracts to create efficient diffractive lenses and beam steerers [125,126,127,128]. As such, diffractive devices are varied in their underlying physics; a brief discussion on the optics of various classes of static diffractive elements is provided. Firstly, a criterion to distinguish between “thin” and “thick” diffractive devices must be made. 

Using the convention of Gaylord and Moharam [129], a “thin” grating exhibits Raman–Nath (RN) type diffraction. The criterion for such is [129],

(18) $$\frac{\pi^{2}d^{2}}{\Lambda_{\mathrm{gr}}^{2}}\frac{\Delta n_{\mathrm{eff}}}{{\bar{n}}_{\mathrm{eff}}cos^{2}\theta }<1$$

where *λ* is the wavelength of light, *d* is device thickness, *Λ*_gr_ is the grating period, *Δn*_eff_ is the maximum change in the refractive index of the material, ${\bar{n}}_{\mathrm{eff}}$ is the average refractive index that light experiences and *θ* is the refraction angle of the wave. For NLCs, where ***n*** fully reorients to create *ϕ*, Equation (18) reduces to approximately,

(19) $$d<\Lambda_{\mathrm{gr}}cos^{2}\theta .$$

Here, typical values of *Δn*_eff_ = 0.2 and ${\bar{n}}_{\mathrm{eff}}=1.6$ have been taken. Raman–Nath (RN) diffraction leads to several diffractive orders, the positions of which obey the grating equation,

(20) $$m\lambda =m\frac{2\pi }{k}=\Lambda_{\mathrm{gr}}sin\theta_{m}=\frac{2\pi }{q_{\mathrm{gr}}}sin\theta_{m}.$$

Here, *m* is the order number, *q*_gr_ is the grating wave number, *k* is light’s wave number, and *θ_m_* is the *m*th order’s diffraction angle. Assuming Fraunhofer diffraction and using the convention of Goodman [125,126], the optical intensity field in the diffraction plane (*U_diff_*) can be found from the Fourier Transform of that on the aperture (*U*_ap_). This is written as

(21) $$U_{diff}\left(x,y\right)=\frac{e^{ikz}e^{\frac{ik}{2z}\left(x^{2}+y^{2}\right)}}{i\lambda z}F\left\{U_{\mathrm{ap}}\left(x^{\prime },y^{\prime }\right)\right\}$$

where F represents the Fourier transform operator. 

**Figure 9:**
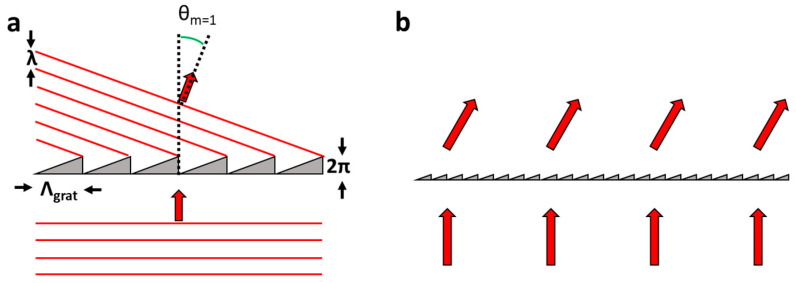
Micro and macroscopic picture of a blazed grating. (**a**) Shows how the 2π phase delay leads to each micro prism modulating the incoming wave to redirect the wavefront, where the angle of redirection is determined by Equation (20). (**b**) Shows the macroscopic effects of this where the optical rays have deviated at a sufficient distance from the device (Fraunhofer diffraction).

For RN phase grating, the shape and magnitude of *ϕ* (here represented in *U*_ap_) determines diffraction efficiency [125,126]. To create efficient RN beam steering components, a *ϕ*, which is a blazed grating with 2π optical resets, is desirable (Figure 9). This is because this structure, in theory, will diffract 100% of light to the *m* = 1, allowing efficient beam steering [130]. Similar blazed phase structures can also be used for highly efficient lensing. These Diffractive Fresnel lenses change either the grating period or amplitude spatially to focus light with 100% efficiency with accurate blazing [131]. 

### 3.2. Bragg Diffraction 

Conversely, a grating can be considered “thick” or of the Bragg type if

(22) $$d>10\Lambda_{\mathrm{gr}}$$

is satisfied [129]. Bragg diffraction physically manifests significantly differently to the RN type. This difference is due to the cause of the diffraction being distinct, where in the Bragg regime, it occurs due to subsequent layers of varying permittivity in the direction of propagation. This effect can be explained by wave-coupling theory [132]. This is relatively complex; however, the result is that the Bragg diffraction patterns tend to have only two orders. The Bragg equation describes the wavelength which Bragg diffraction will occur at

(23) $$m\lambda_{\mathrm{bragg}}=2d_{\mathrm{layer}}sin\theta$$

where *d*_layer_ is the thickness associated with a single layer of modulation and λ_bragg_ is the wavelength at which strong Bragg diffraction will occur. Close to λ_bragg_, the efficiency is close to 100%, depending on the number of layers the waves pass through. 

### 3.3. Diffractive Dielectric Inclusions and Exclusions 

**Figure 10:**
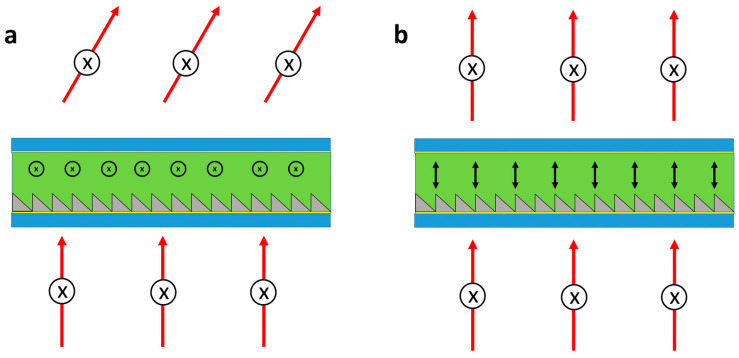
(**a**,**b**) Diagram of the beam deflector device presented by Wang et al., in 2000 [134]. Here, the refractive index of PMMA is approximately equal to n_⏊_ of the NLC, meaning minimal diffraction occurs in the on state.

As with refractive devices, a simple method to create a diffractive beam steering device is through including a static periodic component with the desired diffractive effect due to a contrast in the refractive index with the NLC [133,134]. By applying an external field to reorient the LC optic axis, such devices become switchable optical components. An early example of such a component was demonstrated by Wang et al. in 2000 [134], where a custom-built Poly-Methyl-Meth-Acrylate (PMMA) blazed grating of spatial period 10 μm was placed in a device (shown in Figure 10). This allowed for the switching of the intensity of the *m* = 0 and *m* = 1 orders with the application of a voltage (5% to 85% and 80% to 10% from 0 to 8 V, respectively).

In creating the above devices, several important factors must be considered. Firstly, it is well known that the topography of an enclosing boundary of an NLC adds additional components to the system free energy, which can induce alignment and defects [33,34,48,135,136]. Such effects increase with increasingly deep structures with shorter periods, meaning extensive angles and large contrasts difficult to practically achieve. Secondly, for minimum optical losses, the refractive index of the dielectric inclusion should match that of either *n_e_* or *n_o_*. This has been addressed largely through the design and utilization of the refractive index matching Reactive Mesogen (RM) materials, which offer the dual advantages of the matched refractive index whilst simultaneously acting as an alignment layer for the LC layer [121,133,137]. Thirdly, such devices often have very high driving voltages, due to the dielectric dividing layers put down in the fabrication process (see Section 2.4). 

### 3.4. Single Patterned Electrode Devices 

**Figure 11:**
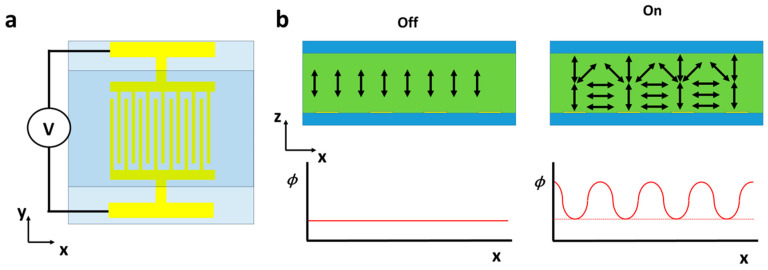
(**a**,**b**) Diagram of a similar device used by Lindquist et al. in 1994 [138]. Here, a voltage is applied to interdigitated electrodes which create a periodic distortion in the NLC. This is passed onto the transmitted light as an optical phase delay (*ϕ*) of the same period, causing switchable diffraction.

An alternative relatively simple method of inducing the required periodic undulation is by utilizing patterned electrodes. An early example of such was performed using simple interdigitated electrodes (IDEs) to create a diffraction grating by Lindquist et al. in 1994 [138] (see Figure 11). The maximum observed efficiencies of *m* = 1 order for this device and other similar ones were found to be around 30% [138,139,140,141], which is comparable to the 38% theoretical limit of a sinusoidal phase grating. Similarly, diffractive microlens arrays have been fabricated with periodically hole-patterned electrodes [142,143]. Here, as the electric field is varied, the diffractive structures change their focus, allowing for potential lensing applications. 

The two fundamental drawbacks in such devices are: firstly, the lack of asymmetry, meaning even at optimized phase contrast their efficiency cannot exceed 40%. Secondly, the period is set by the fabrication process; therefore, such devices can only diffract light to discrete angles rather than being able to vary continuously. Similar to the refractive devices discussed in Section 2.4, an asymmetry can be induced using resistive electrodes [139,144,145]. However, due to the desired feature sizes for diffractive devices being smaller than refractive ones (100 s of μm compared to μm), fabrication becomes substantially more challenging, and hence the devices suffer from the “flyback” region to a greater extent (approximately quantified in Equation (17)). 

### 3.5. Electronically Addressed Diffractive Spatial Light Modulators

If devices are created where the individual electrodes apply an arbitrary voltage, blazed diffractive structures can be incorporated into the LC beam steering devices. This can be achieved using EASLMs. As discussed in Section 2.6, these are limited by the smallest attainable pixel size, the number of grey levels and the flyback region [95,146]. These problems are further compounded in the case of diffractive steering where the smaller feature sizes lead to similar sizes of *w*_feature_ and *w*_fb,_ which reduces the efficiency, particularly for wider angle steering (see Equation (17)) [17,106]. For example, in 2004 a 1D EASLM made up of 2 μm fingers was used to create a 48 μm pitch blazed grating [147]. Here, despite having a relatively small diffraction angle of 0.8° for 633 nm light, a maximum diffraction efficiency of the *m* = 1 order of around 75% was achieved. Recently, values of 99% at 0.1°, 90% at 1° and 25% at 10° were reported [106]. These devices also tend to operate in reflection mode only, due to the complexity of addressing requiring a silicon backplane and the increased *ϕ* due to passing through the NLC twice, which is often required for thin (fast switching; see Equation (12)) EASLMs.

### 3.6. Volume Bragg Gratings

**Figure 12:**
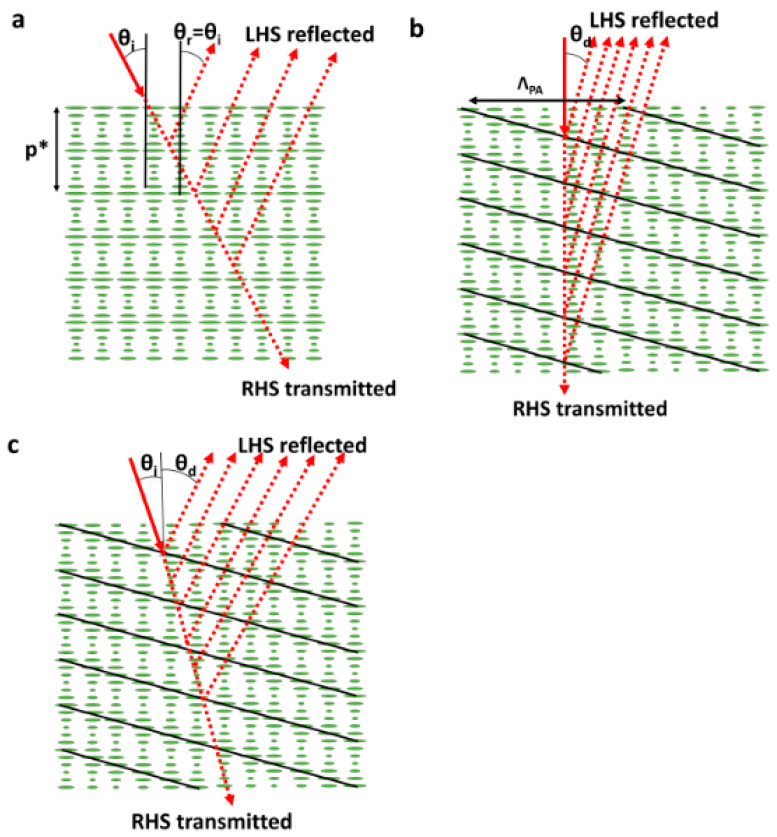
Variations of Volume Bragg Gratings (VBGs). (**a**) Shows simple Bragg diffraction where strong deflection occurs at an angle equal to the incident angle. (**b**) Shows a variation of (**a**) where an offset is induced in the cholesteric pitch such that the entire grating is slanted, allowing normal incidence, with off-normal reflection. (**c**) Demonstrates the effect of off-normal incidence on the tilted grating.

Another liquid crystal class that can be utilized to create wide-angle beam steering devices is cholesteric liquid crystals (N*), as used in Volume Bragg Gratings (VBG). Here, when white light illuminates a Grandjean aligned cholesteric material, close to 100%, reflective Bragg diffraction will occur for circularly polarised light of a certain handedness in a narrow range of wavelengths close to *λ*_Bragg_ (Figure 12a) [16,148,149,150,151,152,153], where,

(24) $$\lambda_{\mathrm{Bragg}}=\frac{n_{e}+n_{o}}{2}p^{*}.$$

where p* is the cholesteric pitch (see Figure 12a). As generally, normal illumination is preferred, this geometry can be altered to the configuration shown in Figure 12b. Here, photoalignment techniques can periodically rotate the director, allowing the Bragg grating to tilt relative to the surface normal. Here, the diffraction angle (*θ_d_*) is determined by the refractive index and the lateral pitch of the photoalignment (*Λ*_pa_),

(25) $$\sin\theta_{d}=\frac{\lambda_{\mathrm{Bragg}}}{n_{o}\Lambda_{\mathrm{pa}}}$$

This can be modified for non-normal incidence (Figure 12c) by adding an off-normal-incidence term [149]

(26) $$\sin\theta_{d}-\sin\theta_{i}=\frac{\lambda_{\mathrm{Bragg}}}{n_{\mathrm{eff}}\Lambda_{\mathrm{pa}}}$$

where *n*_eff_ is the effective refractive index at the incident surface light experiences. 

The main drawback of Bragg devices is that they only reflect strongly within a narrow wavelength range, determined by p*. This means they are inappropriate for continuously steering a monochromatic beam, instead being more suited for applications where a single deflection angle is required. Recently, several mesogens and mixtures have been created where an oblique helicoidal director structure has been achieved [154,155,156,157,158]. One of the key advantages of the oblique helicodal director structure is that the value p* can be continuously varied through an external electric field, preserving a single harmonic heliconical structure [159,160]. 

### 3.7. Diffractive Alignment Gratings 

#### 3.7.1. Diffractive Stacked Alignment Layers 

Similar to that discussed in in Section 2.5, diffractive beam steering devices can be fabricated through NLC alignment. Here, some desired diffractive director profiles are induced by the NLC’s enclosing surfaces, which can then be overwritten in bulk by a large applied voltage. The creation of diffractive blazed gratings has been achieved using SALs to continuously vary alignment pretilt [161,162] or photoalignment to vary the azimuthal or radial angles [41,163]. These devices can have reasonable efficiency—for example, Honma et al., publishing a 100 μm period blazed grating, created using SALs to steer light to the *m* = 1 order (0.19°) with 68% efficiency [161]; however, the angles are clearly small (similar to refractive devices). 

#### 3.7.2. Pancharatnum–Berry Devices 

**Figure 13:**
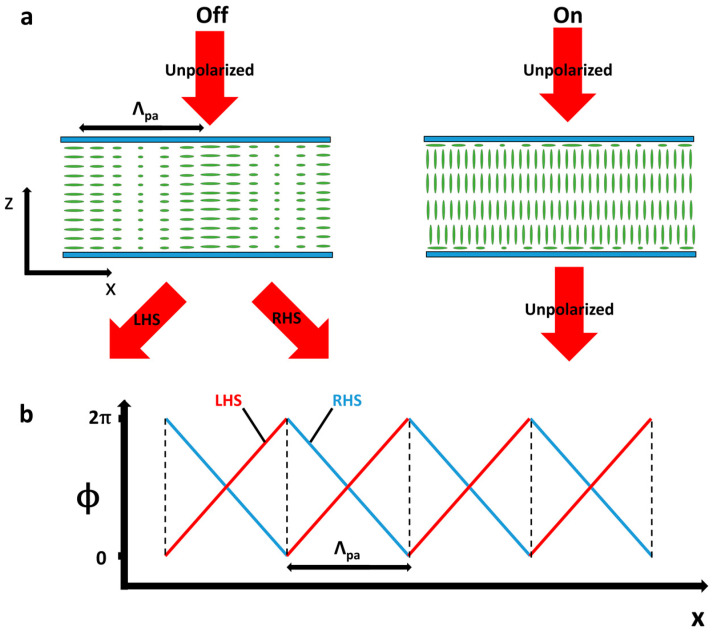
Shows a Pancharatnum–Berry beam deflector. (**a**) Shows the off and on states, respectively, where the light is deflected to the ±1 orders with close to 100% efficiency for each handedness or pass through, respectively. (**b**) Shows the OPD experienced by the two polarisations. LHS and RHS each experience a blazed grating in opposite directions, leading to close to 100% efficiency.

An important sub-class of diffractive alignment gratings are Pancharatnum–Berry (PB) polarisation gratings [163,164]. These PB devices have seen much interest in recent years, and a thorough review of their application in AR and VR technologies was provided by Lee et al. in 2017 [165]. Similar to cholesteric Bragg gratings, shown in Figure 12b, these structures interact differently with the light of different polarisations and can be created by periodically rotating the liquid crystal alignment layer [18,106,165,166,167,168]. The NLC director configuration and mode of operation for these devices are shown in Figure 13a. Here, in contrast to Bragg gratings, it can be seen that the PB devices transmit and deflect light rather than reflect it, and also that they have a standard NLC in contrast to Bragg gratings cholesteric. The optics of this device is described by Jones calculus [21,106,165,166,168], where the cycloidal diffractive waveplate structure induces a 2π optical phase delay for each half director rotation. The result is a device which acts as a perfect (zero-offset) blazed grating for both circularly right and left hand sided components of light, but oriented in opposite directions to one other [165]. In theory, this results in both gratings and lenses being close to 100% diffraction efficiency for each handed input polarization [106,169,170]. In practice, the devices are highly efficient (*η_1/-1_* > 99.5% for relevant input polarisation) and can achieve wide-angle steering (*θ*_d_ > 40°). However, they are limited as their angle of diffraction is determined by the period of the photoalignment (*Λ*_pa_), which cannot be changed in situ as it is set in the fabrication process. 

### 3.8. Flow-Induced Patterns

An alternative method of inducing diffractive structures in NLC is by inducing periodic flow patterns within devices. These flows can then cause the director to experience a torque creating a uniform optical grating. A well-known method of inducing these hydrodynamic gratings is by placing LCs in a state of Electro-Hydrodynamic Instability (EHDI) [47]. Considering the most common EHDI system, a material with negative dielectric anisotropy (*Δε* > 0) and positive conductivity anisotropy (*Δσ* > 0) placed within a homogenous planar aligned device, a wide variety of different textures are formed as both voltage and frequency are varied [171,172,173]. One such is the Normal Roll (NR) texture caused by the director undulating to form an approximately 1D diffraction grating with *Λ*_grat_ of order d [15,174]. Depending on the frequency, the value *Λ*_grat_ can typically vary by a factor of two continuously [171,172,175,176,177]. This typically allows wide-angle low-efficiency steering as the theoretical maximum *η*_*m*=1_ is around 40% without blazing, and significant optical losses occur within the devices. 

### 3.9. Cholesteric Gratings

Another method which can be utilized to create a grating structure is using the natural tendency of a cholesteric NLC to twist in space, where the helical axis aligns parallel to the substrates. This twisting can create a wide range of diffractive textures in which each responds differently to external stimuli, such as temperature, electric fields and optical intensity [170,178,179]. The textures of most interest for diffractive beam steering are the developable modulation (DM) and Growing Modulation (GM) (see Ryabchun et al. for further details [178]). In the DM mode, the grating pitch does not change, but voltage modulation allows for forming a symmetric diffractive structure where the dominant non-zero orders have a maximum efficiency of around 25% [180]. Meanwhile, in the GM mode, *Λ*_grat_ can be varied by a factor of around 1.5 through the application of voltage. Here the intensity also varied with voltage, the values of *η_m_*(*V*) were not reported [180]. 

## 4. Discussion 

### 4.1. Figures of Merit 

The great variety of technologies described in Section 2 and Section 3 make a direct comparison between devices challenging. This is due to each device tending to excel in certain aspects while being more limited in others. In this section, several figures of merit (*f_m_*) are established to overcome these difficulties for making comparisons. These metrics are designed so that a device with ideal properties is indicated by a *f_m_* value of 100%. 

The first figure of merit (*f*_1_) assesses devices for efficiency (*η*), attainable angle (*θ*) and switching times (*τ*),

(27) $$f_{1}=\frac{\eta \sin\theta }{\tau \;\left[ms\right]}$$

*f*_1_, therefore, will be 100% for a device which can steer light with 100% efficiency to a maximum angle of 90° with a ms response time. This procedure is useful for assessing devices for applications where discrete steering to wide angles is required with high speed. 

A second figure of merit, *f*_2_, considers applications where smoothly continuous steering angle with reasonable efficiency is required. It is defined as

(28) $$f_{2}=\eta_{\max}\frac{\Delta \theta_{50}}{90}$$

where *η*_max_ is the maximum efficiency and *Δθ*_50_ is the continuously switchable angle before the efficiency drops to 50% of *η*_max_. Thus, *f*_2_ will equal 100% if beams can be steered from 0° to 90° with only a 50% drop in efficiency. 

The quantity *f*_3_ considers suitability technology for large aperture light deflectors. Applications of such could include smart windows [150,181] and efficient solar harvesting [137,182]. Here,

(29) $$f_{3}=\frac{A_{\max}}{cm^{2}}\eta_{\theta >10}$$

where *A*_max_ is the maximum attained aperture size and *η*_*θ*>10_ is the maximum observed efficiency above 10°.

### 4.2. Refractive NLC Devices 

Table 1 summarises the findings made in assessing refractive NLC beam steering devices. The values *f_m_* were calculated using values given in the supplementary information (Tables S1 and S2). Generally, these devices perform well when the desired application requires continuous and highly efficient steering to relatively small angles, for example, contact lens applications [7,8,9]. The reason most refractive devices struggle to perform wider angle steering is due to too small values of $\frac{\partial n}{\partial x}$. In order to increase deflection power, refractive devices either need to be made thicker (leading to increased losses and reduced switching speed) or introduce Fresnel offsets (leading to increased diffraction reducing efficiency). 

Although most devices discussed are limited in wide-angle steering capability, optical waveguides show excellent properties in both attainable angle and efficiency. Despite these devices having corresponding disadvantages (chiefly their limited aperture size), they demonstrate how significant progress can still be made in refractive LC beam steering through a judicious reimagination of device design. 

### 4.3. Diffractive NLC Devices

Table 2 compares the diffractive LC beam steering devices for *f_n_* calculations. Clearly, for such devices, larger angles are consistently possible compared to those operating under refractive principles. The central challenge with diffractive NLC beam steering devices is maintaining high efficiencies while continuously modifying the resultant steering angles. The majority of devices with the highest efficiency (Dielectric Inclusions, SAL, PB and VBG) are all fixed in diffraction angles, significantly limiting their flexibility. Meanwhile, the diffractive efficiency of devices that can continuously vary pitch (EHDI, DM, photoconductive gratings) have significantly lower efficiencies, mainly due to the difficulty incorporating a blaze into the phase profile of transmitted light. At present, the best option for relatively high efficiency and continuous steering is the LCOS systems, which can be both efficient and continuous in steering to small angles. In such systems, the current major limitation is the difficulty in forming perfect 2π offsets in the phase profile, due to the finite size of the flyback regions. This can be further improved utilizing either liquid crystal materials with further tailored electronic and elastic properties or designing more advanced driving circuitry. 

### 4.4. Non-Nematic Liquid Crystal Devices

To this point, we have considered only nematic LCs. Although they are most commonly utilized to create LC beamsteering devices, there has also been significant progress in creating devices with other LC phases. This section will briefly discuss a few examples of how these novel phases have been applied in beam steering. 

#### 4.4.1. Isotropic LC Phases 

A major limitation of using NLCs in beam steering devices is their inherent polarization dependence. Although this can be overcome, to an extent, through judicious design (see Section 2.9), it remains a significant technical problem. A potential solution for this is to utilize more novel optically isotropic LC phases which change their optical properties when an electric field is applied (for example, due to the Pockels or Kerr effect). Such phases include blue phases [185,186,187], the Dark Conglomerate (DC) phase [188,189,190] and polymer nano-dispersed liquid crystals (PDLCs) [191,192]. However, these phases also tend to have disadvantages compared to NLCs, including the degradation of materials, elevated transition temperatures, narrow phase temperature ranges and more limited knowledge of the phases generally. Many of these issues are addressable with further research. For example, the widening of the phase range and reduction in *T*_NI_ in blue phase materials [193,194], has led to significant progress in creating polarization-independent optical elements [195,196,197,198,199,200,201]. Although such devices are expected to have high driving voltages, polarization independence dramatically increases efficiency for unpolarized light. In a recent paper by Tian et al. [199], a blue phase device with dielectric inclusion was proposed and modelled, where continuous deflection from 0° to 1.7° of both polarisations was predicted. Similarly, a geometric lens made from the DC phase was predicted to continuously vary focus from 12 mm to ∞ in a polarisation independent manner [188]. 

#### 4.4.2. Nematic Twist Bend 

Another phase of interest in recent years is the Twist Bend Nematic (N_TB_) [155,156,158,202,203,204,205]. As the N_TB_ phase forms relatively periodic optical structures, there has been interest in using them for the application of diffractive beam steering devices [206,207,208]. Such devices are advantageous due to short pitch optical structures; however, they have reported low relative efficiencies ($\xi_{m\neq 0}<15\%)$ and a pitch that is determined by temperature [206]. Further investigation of the N_TB_ phase may lead to more promising results, specifically if they are similar to VBG’s, but the temperature dependent pitch could be converted to being controlled by the electric field [160]. 

#### 4.4.3. Ferroelectric LCs

Another set of materials, which have played a main role in LC beam steering devices, are ferroelectric liquid crystals (FLCs). These utilize the ferroelectric properties of surface stabilized chiral smectic C phase (SmC*) [209,210,211]. Ferroelectric LCs have been of interest for displays and SLMs for many years [212], mainly due to their faster switching speeds compared to NLCs (sub 100 μs). The ability to switch faster is also crucial to beam steering applications [213], as it allows the use of Frame Sequential Colour switching [214,215]. Here, the light source strobes between three primary colours, each of which will experience a different customized phase profile and then switch before the next colour. The human eye cannot keep up with the strobe and interprets the transmitted light from such devices as full-colour holograms. Historically, the drawbacks of using FLCs comes from the difficulty of aligning and maintaining the SmC* configuration, making them particularly prone to irreversible damage when subjected to elastic shock [216]. This is still an active research area with, for example, surface relief gratings being utilized to stabilise the SmC* phase [217]. Due to the difficulty of using SmC* phases, the recent discovery of a polar nematic phase is of particular note for LC beam steering [218,219]. If such materials are successfully applied to beam steering devices, switching times may dramatically reduce, increasing viability for high-speed applications. 

### 4.5. Summary 

In summary, the field of LC beam steering has developed dramatically from initial concepts and ideas into a mature and advanced research area in the last forty years. With the range of methodologies discussed, it should be clear that many adaptive optical components can be achieved using LCs, and the exact mode of operation mainly depends on the proposed application. In the case of unpolarized light, significant limitations in the field remain in achieving wide aperture, wide-angle, high efficiency and continuous beam steering devices, and in removing the polarisation dependency that limits devices. Future developments are likely to seek to address these issues, where innovations are likely to come from the complementary judicious design of both the required device architectures and materials to overcome these limitations. 

## Figures and Tables

**Table 1:** Summary of advantages and disadvantages of refractive devices.

Device Type	Key Advantages	Key Disadvantages	*fn* (%)		
			*f*_1_	*f*_2_	*f*_3_
Geometric Prisms and Lenses [19,58]	Simple Fabrication High *η* (80%)	Low *θ* (1°) High *d*	0.1	0.9	0
Fringing Field Refraction Devices [60]	Simple Fabrication High *η* (80%)	Low *θ* (0.1°)	0.001	0.1	0
Alignment Prisms/Lenses [88]	High *η* (80%)	Low *θ* (1°) Complex Fabrication	0.02	0.2	0
Refractive EASLM [106,183]	High *η* at small angles (99% at 0.1°)	Reflection only using LCOS *η* decreases with *θ* (90% at 1°)	0.2	7	0
Optical Waveguides [120]	High *η* (80%) High *θ* (40°) Continuous Steering	Small A (20 μm × 1 cm) Not flat-panel	500	40	0.3

**Table 2:** Summary of advantages and disadvantages of diffractive devices.

Device Type	Key Advantages	Key Disadvantages	*f_n_* (%)		
			*f*_1_	*f*_2_	*f*_3_
Dielectric Inclusions and Exclusions [134]	High *η* (80%) High *θ* (10°)	Discreet Diffraction High Voltage	1	0	80
Single Patterned Electrode [184]	High *θ* (10°)	Low *η* (30%)	3	0	30
Diffractive EASLM [106,183]	High *η* at small angles (90% at 1°)	*η* decreases at wide angles (25% at 10°)	0.4	7	30
SAL Gratings [161]	Reasonable *η* (68%)	Small *θ* (0.2°)	0.02	0	0
Photoconductive gratings [111]	Reasonable *θ* (2°)	Low *η* (35%)	0.0001	1	0
PB gratings [165]	Excellent *η* (99.5%) Wide-angle steering (40°)	Discrete Steering Angles	60	0	100
EHDI Gratings [176]	Continuously Variable Reasonable *θ* (8°)	Low *η* (15%) Material Degradation	0.002	0.7	4
VBGs [151,153]	High *η* (90%) High *θ* (50°)	Reflection only	3	0	90
N* GM E-field [178]	Reasonable *θ* (7°)	Low *η* (25%)	0.03	N/A	N/A
N* DM E-field [178]	Reasonable *θ* (12°) Continuously Variable	Low *η* (15%)	0.03	0	N/A

## Supplementary Materials

The following are available online at https://www.mdpi.com/2072-666X/12/3/247/s1. Table S1: Calculations of figures of merit (fm) in refractive devices. Here, if a parameter (x) tends to increase or reduce with another parameter (y), column x includes ~y or ~1/y, respectively. These are first order approximations and are frequently not direct power laws; Table S2: Calculations of figures of merit (fm) in diffractive devices. The proportionality between parameters is shown in the same fashion as in Table S1.
