# Supplementary material for: Liquid Crystal Devices for Beam Steering Applications
Source: Micromachines (Basel). 2021 Feb 28;12(3):247. doi: 10.3390/mi12030247 (PMC7997246; doi:10.3390/mi12030247)
Supplement: Supplementary file 1 [file micromachines-12-00247-s001.pdf]

micromachines

Review

Liquid Crystal Devices for Beam Steering Applications

Rowan Morris, J. Cliff Jones and Mamatha Nagaraj *

School of Physics and Astronomy, University of Leeds, Leeds LS2 9JT, UK; py13rm@leeds.ac.uk (R.M.); j.c.jones@leeds.ac.uk (J.C.J.)
* Correspondence: m.nagaraj@leeds.ac.uk; Tel.: +44-113-3438475

Supplementary Information on Calculations of Figures of Merit

Section 4.1 introduces three figures of merit ($f_{m=1\text{-}3}$). The values of the components f_m for refractive and diffractive devices are given in Table S1 and Table S2, respectively. Given the breadth of literature on the topic, specific articles were chosen to represent techniques where the aim was to show optimized examples of the operating techniques. It was frequently impossible to find precise values for the various parameters, which led to estimates being made. These are marked with an asterisk (*) and were generally obtained through considering other devices where the parameter would be similar or within range. In researching, it was particularly difficult to find values of A_{max} for beam steering devices. For this reason, the value 1 cm^2 was chosen as both the default and maximum choice, as this is standard for an LC device. The literature was then searched to find significant deviations from this value, and these were added where found.

Table S1. Calculations of figures of merit (f_m) in refractive devices. Here, if a parameter (x) tends to increase or reduce with another parameter (y), column x includes ~y or ~$\frac{1}{y}$, respectively. These are first order approximations and are frequently not direct power laws.

Device Type	f_1			f_2		f_3	
Parameters	η (%)	θ (°)	τ (ms)	η_{max} (%)	$\Delta\theta_{50}$ (°)	A_{max} (cm^2)	$\eta_{\theta>10}$ (%)
Geometric Prisms and Lenses [1,2]	80*<br>~$\frac{1}{d}$	1<br>~d	10*<br>~d	80*<br>~$\frac{1}{d}$	1<br>~d	1*	0
Fringing Field Refraction Devices [3]	80*<br>~$1/d$	0.1<br>~d	100*<br>~d	80*	0.2	0.01*<br>~$1/\eta$	0*
Alignment Prisms/Lenses [4]	80*	0.2	10*	80*	0.2	1	0
Reflective EASLM[5,6]	99<br>90<br>25<br>~$1/\theta$	0.1<br>1<br>10<br>~$1/\eta$	10	99	6	1	0
Optical Waveguides [7]	80	40	0.1	80	40	0.004<br>(20 µm × 2 cm)	80

micromachines

Table S2. Calculations of figures of merit (f_m) in diffractive devices. The proportionality between parameters is shown in the same fashion as in Table S1.

**Device Type**	f_1			f_2		f_3	
Parameters	η **(%)**	θ **(°)**	τ **(ms)**	η_{max} **(%)**	$\Delta\theta_{50}$ (°)	A_{max} **(cm²)**	$\eta_{\theta>10}$ **(%)**
Dielectric Inclusions and Exclusions [8]	80	10	10<br>~*d*	80	0	1	80
Single Patterned Electrode [9]	30	10*	2	30	0	1	30
Diffractive EASLM[5,6]	90<br>25<br>~1/ θ	1<br>10<br>~1/η	10	99	6	1	25
SAL Gratings [10]	68	0.2	10*	68	0	0.01*	0
Photoconductive gratings [11]	35	2*	10000	35	2*	1*	0
PB gratings [12]	100	40	1	100	0	1	100
EHDI Gratings [13]	15<br>~1/*d*	8<br>~1/*d*	1000	15	4	1	4
VBGs [12,14]	90	50	20	90	0	1	90
N* GM E-field [15]	25	7	100*	N/A	3	1*	N/A
N* DM E-field [15]	15*	12	100*	25	0	1*	N/A

micromachines

References

1. Love, G.D.; Major, J. V.; Purvis, A. Liquid-crystal prisms for tip-tilt adaptive optics. *Opt. Lett.* **1994**, *19*, 1170, doi:10.1364/ol.19.001170.
2. Titus, C.M.; Bos, P.J.; Lavrentovich, O.D. Efficient accurate liquid crystal digital light deflector. In Proceedings of the Diffractive and Holographic Technologies, Systems, and Spatial Light Modulators VI; Cindrich, I., Lee, S.H., Sutherland, R.L., Eds.; 1999; pp. 244–253.
3. Ye, M.; Wang, B.; Sato, S. Study of liquid crystal lens with focus movable in focal plane by wave front analysis. *Japanese J. Appl. Physics, Part 1 Regul. Pap. Short Notes Rev. Pap.* 2006, *45*, 6320–6322.
4. Bezruchenko, V.S.; Muravsky, A.A.; Murauski, A.A.; Stankevich, A.I.; Mahilny, U. V. Tunable Liquid Crystal Lens Based on Pretilt Angle Gradient Alignment. *Mol. Cryst. Liq. Cryst.* **2016**, *626*, 222–228, doi:10.1080/15421406.2015.1106890.
5. McManamon, P.F.; Bos, P.J.; Escuti, M.J.; Heikenfeld, J.; Serati, S.; Xie, H.; Watson, E.A. A Review of Phased Array Steering for Narrow-Band Electrooptical Systems. In Proceedings of the Proceedings of the IEEE; 2009; Vol. 97, pp. 1078–1096.
6. Thalhammer, G.; Bowman, R.W.; Love, G.D.; Padgett, M.J.; Ritsch-Marte, M. Speeding up liquid crystal SLMs using overdrive with phase change reduction. *Opt. Express* **2013**, *21*, 1779, doi:10.1364/oe.21.001779.
7. Davis, S.R.; Farca, G.; Rommel, S.D.; Johnson, S.; Anderson, M.H. Liquid crystal waveguides: new devices enabled by >1000 waves of optical phase control.; Chien, L.-C., Ed.; 2010; p. 76180E.
8. Wang, X.; Wilson, D.; Muller, R.; Maker, P.; Psaltis, D. Liquid-crystal blazed-grating beam deflector. *Appl. Opt.* **2000**, *39*, 6545, doi:10.1364/AO.39.006545.
9. Li, G.; Mathine, D.L.; Valley, P.; Äyräs, P.; Haddock, J.N.; Giridhar, M.S.; Williby, G.; Schwiegerling, J.; Meredith, G.R.; Kippelen, B.; et al. Switchable electro-optic diffractive lens with high efficiency for ophthalmic applications. *Proc. Natl. Acad. Sci. U. S. A.* **2006**, *103*, 6100–6104, doi:10.1073/pnas.0600850103.
10. Honma, M.; Nose, T.; Yanase, S.; Yamaguchi, R.; Sato, S. Liquid-Crystal Blazed Gratings with Spatially Distributed Pretilt Angle. *Jpn. J. Appl. Phys.* **2010**, *49*, 061701, doi:10.1143/JJAP.49.061701.
11. Lin, S.-H.; Huang, B.-Y.; Li, C.-Y.; Yu, K.-Y.; Chen, J.-L.; Kuo, C.-T. Electrically and optically tunable Fresnel lens in a liquid crystal cell with a rewritable photoconductive layer. *Opt. Mater. Express* **2016**, *6*, 2229, doi:10.1364/OME.6.002229.
12. Lee, Y.-H.; Tan, G.; Zhan, T.; Weng, Y.; Liu, G.; Gou, F.; Peng, F.; Tabiryan, N. V; Gauza, S.; Wu, S.-T. Recent progress in Pancharatnam-Berry phase optical elements and the applications for virtual/augmented realities. **2017**, doi:10.1515/odps-2017-0010.
13. Morris, R.; Jones, J.C.; Nagaraj, M. Continuously variable diffraction gratings using electroconvection in liquid crystals for beam steering applications. *J. Appl. Phys.* **2019**, *126*, 224505, doi:10.1063/1.5128205.
14. Denisov, A.; De Bougrenet De La Tocnaye, J.L. Resonant gratings in planar Grandjean cholesteric composite liquid crystals. *Appl. Opt.* **2007**, *46*, 6680–6687, doi:10.1364/AO.46.006680.
15 Ryabchun, A.; Yakovlev, D.; Bobrovsky, A.; Katsonis, N. Dynamic Diffractive Patterns in Helix-Inverting Cholesteric Liquid Crystals. *ACS Appl. Mater. Interfaces* **2019**, *11*, 10895–10904, doi:10.1021/acsami.8b22465.
